# Psychiatrists’ perceptions of the clinical importance, assessment and management of patient functioning in schizophrenia in Europe, the Middle East and Africa

**DOI:** 10.1186/1744-859X-12-8

**Published:** 2013-03-26

**Authors:** Philip Gorwood, Tom Burns, Georg Juckel, Alessandro Rossi, Luis San, Ludger Hargarter, Andreas Schreiner

**Affiliations:** 1 CMME, Sainte-Anne Hospital, Paris-Descartes University, 100 rue de la Santé, Paris, Cedex 14, 75674, France; 2 INSERM UMR894, Centre of Psychiatry and Neuroscience, 2ter rue d'Alesia, Paris 75014, France; 3 Department of Psychiatry, Warneford Hospital, University of Oxford, Oxford, OX3 7JX, UK; 4 Psychiatrie, LWL-Universitaetsklinikum der Ruhr-Universitaet Bochum, Bochum, 44791, Germany; 5 Department of Experimental Medicine, University de L'Aquila, Coppito II, L'Aquila, 67100, Italy; 6 Hospital Sant Joan de Déu, Centro de Investigación Biomédica en Red de Salud Mental (CIBERSAM), Passeig Sant Joan de Déu 2, Esplugues de Llobregat, Barcelona, 08950, Spain; 7 Department of Medical and Scientific Affairs, Janssen EMEA, Johnson & Johnson Platz 1, Neuss, 41470, Germany

**Keywords:** Assessment, Functioning, Management, Psychiatrist, Schizophrenia, Survey

## Abstract

**Background:**

It has been estimated that as many as two thirds of patients with schizophrenia are unable to perform basic personal and social roles or activities. Occupational functioning and social functioning, as well as independent living, are considered as core domains of patient functioning. Improvement in patient functioning has also been recognized as an important treatment goal in guidelines and an important outcome by regulatory agencies. Nevertheless, information is lacking on how these aspects are being considered by psychiatrists across the world and how they are being assessed and managed.

**Methods:**

The ‘Europe, the Middle East and Africa functioning survey’ was designed to canvas opinions of psychiatrists across these regions to ascertain their perceptions of the clinical importance, assessment and management of functioning amongst their patients with schizophrenia. The survey comprised 17 questions and was conducted from March to April 2011 in 42 countries. Data collected included the demographics of respondents and their opinions regarding personal and social functioning in patients with schizophrenia.

**Results:**

Results were obtained from 4,163 clinicians. Psychiatrists estimated that more than two thirds (70%) of their patients with schizophrenia showed impaired or very poor levels of functioning. The majority of psychiatrists (92%) believed that personal and social functioning was an important treatment goal for patients with schizophrenia, and 91% believed it was an important goal for patients’ families. The majority of psychiatrists (55%) assess the personal and social functioning of their patient at each visit; however, 81% reported that they determine the level of functioning through clinical interview and not by using a specific assessment scale. To manage personal and social functioning in their patients, 26% of psychiatrists prefer pharmacological interventions, whereas 46% prefer psychosocial interventions.

**Conclusion:**

Psychiatrists recognize that functioning is impaired/very poor in patients with schizophrenia, and there is still an important need to address functioning as a main treatment goal for patients with schizophrenia.

## Background

Schizophrenia is a complex and heterogeneous disorder, with a range of symptoms and effects on the lives of patients and their families [1,2]. It impacts to a significant and detrimental extent on patient functioning; indeed, this deficit in functioning is recognized as a diagnostic criterion of schizophrenia [3]. Several domains of functioning are affected, including occupational and social aspects and independent living [4,5].

Social functioning, in particular, is widely recognized as an important factor when considering long-term outcomes in patients with schizophrenia [6-8]. In one study of patients in six European centres, impairments in social functioning were observed in over 78% of the population at assessment within 2 years of first-episode psychosis, and this persisted at subsequent 1-, 2- and 15-year follow-ups [9]. Up to two thirds of patients are unable to perform basic social roles, even when psychotic symptoms are in remission; only a minority marry, and less than one third are in regular employment [10]. There are regional differences in employment rates amongst patients with schizophrenia that may be linked in part to social or environmental influences such as local welfare or benefit systems, legislative and economic factors [11]. A US study found the number of patients with schizophrenia in regular, paid employment to be less than 15%. In addition, unemployed patients presented with lower quality of life scores, and an inverse relationship existed between the likelihood of employment and the receipt of disability payments [12]. Poor functioning therefore places a considerable burden (including an economic impact) on society [8,10].

Improvement in functioning in patients with schizophrenia is now considered to be an important treatment goal [13-15]. As such, it is included in treatment guidelines [1,8,16] and has been recognized as a useful endpoint in the evaluation of treatment options by regulatory authorities [17]. A recent literature review highlighted an increase in the number of studies documenting symptomatic remission and an awareness of functioning and quality of life as outcome measures [18]. A number of studies have reported improved patient functioning following treatment with antipsychotics [19-24] or with non-pharmacological interventions such as social skills training, cognitive behavioural therapy, cognitive therapy, cognitive remediation and social cognition training [25-27]. Improved social and occupational functioning is also identified by patients and their families as an important treatment outcome [10], as well as by groups representing patients [6].

The remission of positive and negative symptoms does not always correspond with an improvement in functioning [6,15,28,29], and improvements in symptoms do not always correlate with patient satisfaction or with performance in social relationships or performance of daily activities [30]. In one study, 3 years after beginning initial antipsychotic treatment, symptomatic remission rates were 60.3%, functional remission rates (defined by employment status, social relationships and independent living) were 45.4%, and 57% of patients achieved adequate subjective well-being. However, only 28.1% achieved remission in all three areas [31]. Social functioning may, therefore, represent an area that requires treatment beyond the resolution of overt symptoms of psychosis [32]. Conversely, early improvements in social functioning [33] or subjective well-being may be predictors of symptomatic remission [34] and of a good overall treatment outcome, supporting the importance of assessing social functioning in patients with schizophrenia [33]. Methods of gathering information in order to assess functioning are, however, known to vary and can include the perceptions or ratings of health care professionals, patients and family members [10,35]. While a number of assessment scales are used, including the Social and Occupational Functioning Assessment Scale (SOFAS) [36], the Functional Remission Of General Schizophrenia (FROGS) scale [37], the Global Assessment of Functioning (GAF) scale [38] and the Personal and Social Performance (PSP) scale [39], there is a lack of standardization in methodology, as highlighted by several authors [6,7,10,13,40].

Improved social functioning is an important treatment goal for patients with schizophrenia which does not necessarily correlate with improvement in disease symptoms. Despite the prevalence of impaired functioning, assessments of functional outcome are neither routinely conducted nor effectively standardized in clinical practice. As such, some authors recommend a change in current attitudes and perceptions with respect to the importance, evaluation and treatment of impairments in functioning [32]. Results from a survey previously conducted in Spain designed to assess the opinions and perceptions regarding functioning in patients with schizophrenia highlighted a need to obtain a broader understanding of the assessment of the measurement and treatment of patient functioning [41]. Results showed that psychiatrists considered the control of psychotic symptoms as the most important treatment objective, with outcomes such as functioning and relapse prevention regarded as secondary therapeutic objectives. Ninety-two percent of psychiatrists considered that functioning should be recorded in medical notes; only 17% of the psychiatrists surveyed used specific scales to assess their patients' functioning. Thirty-three percent of respondents thought that <20% of their patients had an adequate level of functioning, and 76% considered that patient functioning would be best improved by modifying pharmacological treatment. Overall, this survey showed that clinicians' perception of the level of functioning in their patients with schizophrenia was low and that there is a need to develop protocols to improve patient functioning [41]. How this type of perception applies in other countries (from Europe and elsewhere) is an important consideration in relation to improving the following: (1) the quality of assessment of patient functioning by psychiatrists, (2) the frequency of appropriate assessments of the patients' autonomy and (3) the knowledge and use of available therapeutic techniques that facilitate such processes.

The present survey was performed across countries in Europe, the Middle East and Africa, and aimed to determine psychiatrists' perceptions of the importance of social functioning in their patients, the ways in which deficits are assessed and managed and potential barriers to improving social functioning in patients with schizophrenia.

## Methods

This study aimed to investigate psychiatrists' opinions and perceptions of functioning in their patients with schizophrenia. A paper-based survey was conducted amongst psychiatrists (and neurologists with a psychiatric background in Germany) treating patients with schizophrenia in 42 countries, between March and April 2011, to document the management of social functioning across Europe, the Middle East and Africa.

A scientific committee of international experts with a special interest in the functioning of patients with schizophrenia developed a survey specifically to conduct in Europe, the Middle East and Africa to understand further psychiatrists’ opinions and perceptions of functioning in patients with schizophrenia. The survey consisted of 17 questions, 4 relating to the demographic profile of respondents and the remaining 13 regarding the extent of impaired functioning seen in clinical practice, the role of personal and social functioning as a treatment goal, and approaches to the assessment and management of personal and social functioning in patients with schizophrenia. Finalization of the questions, their wording and the approach to analysis of the survey results took place during a series of steering committee meetings, funded by Janssen, and with input from an independent company with prior experience in conducting surveys of the perceptions of health care providers.

In order to maximize the reach of the survey and to capture any potential geographical variations regarding the perceptions and opinions of functioning in patients with schizophrenia, psychiatrists were identified using a database held by Janssen. Surveys were delivered by mail (in Belgium, The Netherlands, Germany, Serbia, Spain, France, UK and Switzerland) or by Janssen representatives (in other countries). Psychiatrists received a sealed pack containing the survey, a prepaid envelope and a signed letter from the steering committee explaining the aims of the survey. The survey was translated from English into local languages when required. The survey took approximately 10 min to complete. Surveys were completed anonymously, and no individual patient data were collected. Respondents to the survey provided informed consent for the use of the results for research purposes. A copy of the consent form is available for review by the Editor-in-Chief of this journal. Completed surveys were returned directly and blinded to an independent third party for data analysis. The survey was not designed to allow statistical analysis of the differences in responses. Interpretation of the results reported here is based on qualitative comparison of the responses obtained.

## Results

### Respondent demographics

Out of 31,570 surveys distributed, 4,163 were returned from 42 countries in Europe, the Middle East and Africa (Additional file 1) which reflects a response rate of 13%. Fifty-two percent of respondents were male, and 39% were female (9% did not respond to this question). Most (62%) had 10 or more years' experience in schizophrenia, and 30% had less than 10 years' experience (8% did not respond to this question). Of those who responded to specific questions regarding their place of work, 53% of respondents worked in academic settings, 74% indicated that their workplaces received public funding and 51% indicated that their workplaces had both inpatient and outpatient facilities (Additional file 2).

### Extent of functioning impairments in patients with schizophrenia

**Figure 1:**
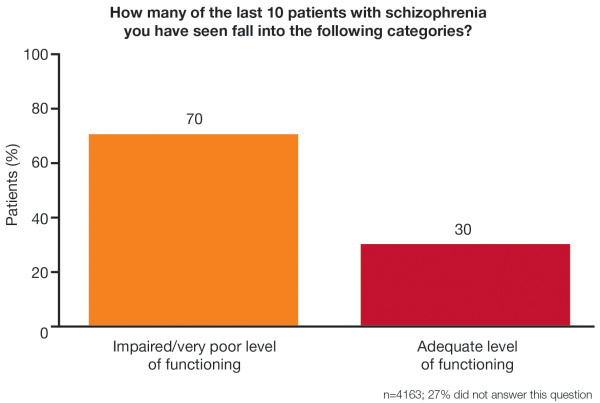
The level of functioning in patients with schizophrenia, as estimated by psychiatrists.

Based on an assessment of the last ten patients seen with an established diagnosis of schizophrenia, psychiatrists estimated the number of patients with adequate, impaired or very poor levels of functioning. An average of 70% of patients were judged to have impaired or very poor levels of functioning (Figure 1). This view was broadly shared across all responders from Europe, the Middle East and Africa (mean values ranged between countries from 55% to 81%; data not presented here).

### Importance of personal and social functioning as a treatment goal

**Figure 2:**
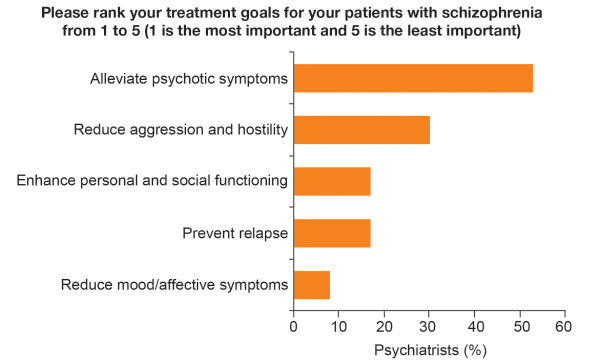
Psychiatrists' perceptions of the most important treatment goals for patients with schizophrenia.

Based on a listing of response options of the most important treatment goals identified by the scientific committee, 53% of psychiatrists ranked ‘alleviate psychotic symptoms’ as the most important treatment goal (Figure 2). Enhancing personal and social functioning was considered the most important treatment goal by 17% of psychiatrists treating patients with schizophrenia (Figure 2), ranking third along with prevention of relapse.

Psychiatrists were asked to indicate their level of agreement regarding the importance of social functioning as a treatment goal both for their patients and for the families of patients with schizophrenia. Ninety-two percent of psychiatrists agreed or strongly agreed that social functioning is an important treatment goal for patients with schizophrenia, and 91% agreed or strongly agreed that this is also an important goal for the families of patients.

### Assessment of personal and social functioning

To assess further how psychiatrists measured personal and social functioning, respondents were asked whether a patient's level of social functioning should be measured regularly and recorded. The majority of psychiatrists (84%) agreed or strongly agreed that a patient's level of social functioning should be measured regularly and recorded. Fifty-six percent of psychiatrists assessed personal and social functioning in patients with schizophrenia at every visit, 25% assessed functioning every two to three visits, 12% assessed functioning two to three times per year, 2% never assess or record functioning and 5% did not respond (Additional file 3).

In order to determine how psychiatrists assessed personal and social functioning in their patients, respondents were required to select from a list that included assessment scales they used. The scientific committee considered that PSP, GAF and SOFAS were the most relevant scales and the most important in clinical use, and as such were used as the three options in this survey. When asked which approach was most often used to assess a patient's level of personal and social functioning, the majority of psychiatrists surveyed (81%) used clinical interviews. When asked specifically which scales they used most frequently, 10% of psychiatrists used functioning scales, with 47% of these preferring to use the GAF scale, 25% preferring to use the PSP scale and 9% preferring to use the SOFAS scale. The majority of psychiatrists (87%) agreed or strongly agreed that obtaining a family member's opinion on the patient's level of social functioning was important (Additional file 3).

In asking respondents whether it was clinically useful to distinguish between improvement in symptoms and improvement in social functioning, the majority of psychiatrists (85%) agreed or strongly agreed that it was clinically relevant to distinguish between these clinical characteristics (Additional file 3). Fifty-six percent of respondents agreed or strongly agreed that cognitive impairment and social functioning should be assessed as two separate entities in patients with schizophrenia, although 20% disagreed or strongly disagreed with treating cognitive impairment and social functioning as separate entities (Additional file 3). Sixty-seven percent of participants agreed or strongly agreed that a consensus or guideline on patient and social functioning would be a useful tool in clinical practice.

### Improvement of personal and social functioning

When asked during which stage of schizophrenia do they focus most to improve their patients' social functioning, the majority of psychiatrists (84%) focused most on improving social functioning in their patients either within 6 months of the onset of the illness or within 5 years of diagnosis (Additional file 3).

**Figure 3:**
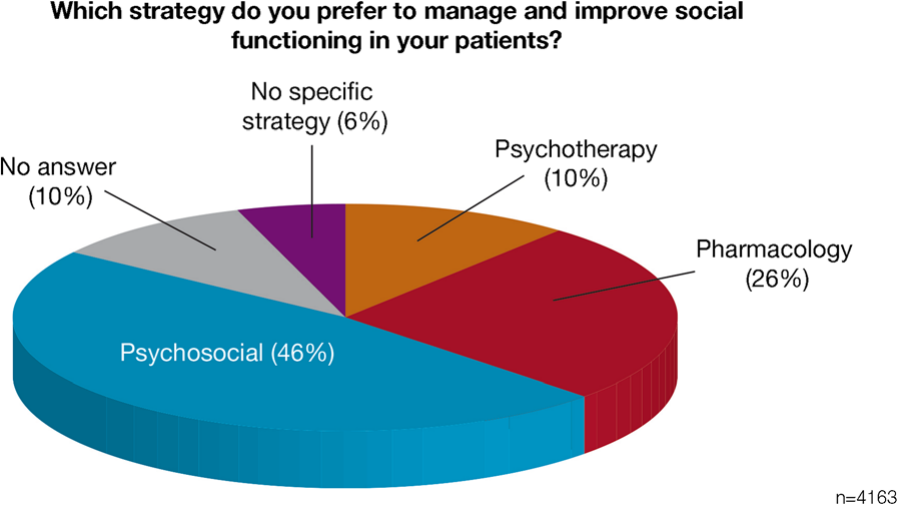
**Psychiatrists**' **preferred strategies for managing and improving social functioning.**

Upon detection of a low level of social functioning in patients with schizophrenia, the first step most commonly taken by psychiatrists (41%) was to use a psychosocial intervention, whereas many (34%) would reconsider the patient's current drug therapy (Additional file 3). The scientific committee viewed psychotherapy, pharmacological treatment and the utilization of a psychosocial intervention (e.g. social, psychological, psychoeducation) as the most relevant strategies to manage and improve social functioning in patients with schizophrenia. Forty-six percent of respondents preferred to use psychosocial strategies to manage and improve social functioning, while 26% favoured pharmacological strategies and 10% favoured psychotherapy (Figure 3). Of the 4,163 psychiatrists surveyed, 6% did not employ a specific strategy to improve social functioning, and 10% did not answer this question (Figure 3).

When asked how psychiatrists aimed to improve the social functioning of their patients with schizophrenia, 47% of respondents reported that they most often ask a member of the patient's family for support in improving their patient's social functioning, whereas others (40%) would seek the input of another health care professionals (HCPs) within the multidisciplinary team (Additional file 3).

**Figure 4:**
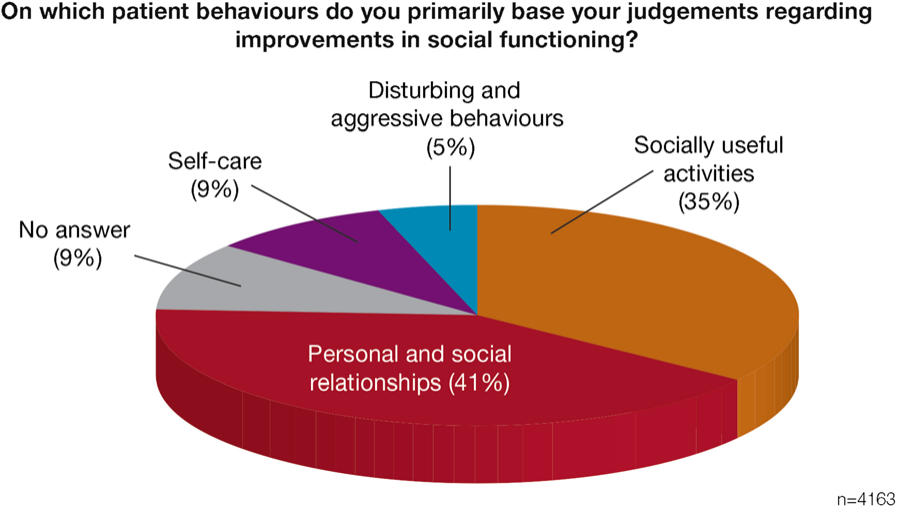
The patient behaviours on which psychiatrists primarily based judgements regarding improvements in social functioning.

Following this, psychiatrists were asked on which patient behaviours they primarily based their judgments regarding improvements in social functioning. Improvements were most commonly judged by changes in the patient's personal and social relationships (41% of respondents) or by engagement in socially useful activities, such as work or study (35%) (Figure 4).

### Pharmacological strategies for improving personal and social functioning

Altering the dose of the current antipsychotic was the preferred pharmacological strategy used to address deficits in social functioning (used by 35% of respondents) followed by switching to another antipsychotic agent (27%) or switching to another formulation (12%). The addition of antidepressants (7%), another antipsychotic (5%) or mood stabilizers (3%) was the least frequently applied pharmacological strategy used by the psychiatrists surveyed to address social functioning. There was a strong agreement amongst the psychiatrists surveyed that the most important factors in selecting a pharmacological agent to improve social functioning were efficacy and tolerability (75% and 56% of respondents ranked this as ‘very important,’ respectively) (Additional file 3).

## Discussion

This survey gathered information on the perceptions and opinions of psychiatrists across Europe, the Middle East and Africa regarding the personal and social functioning of patients with schizophrenia. The survey demonstrated that a large proportion of patients with schizophrenia were considered by their clinician as having impaired personal and social functioning and that the vast majority of psychiatrists believed that it was important to assess functioning on a regular basis.

This survey found that while psychiatrists believe improving personal and social functioning to be an important treatment goal for patients and their families, they ranked this behind ‘alleviating psychotic symptoms’ and ‘reducing aggression and hostility’ when asked to rank the treatment goals for their patients with schizophrenia. While symptomatic remission has been cited as an important precursor to functional remission [18,42], it does not, however, guarantee improvements in functioning. Rates of symptom remission normally exceed those of good functional outcome [15]. A recent study of outpatients with schizophrenia concluded that while almost half were in symptomatic remission, only 10% had attained adequate social and/or vocational functioning [29]. Similarly, an open-label antipsychotic trial found that remission status did not correlate with improvements in positive or negative symptoms or in cognitive functioning [43]. Considered alongside the growing body of evidence that alleviation of symptoms may not correlate with improvements in social functioning in patients with schizophrenia, the findings of this survey suggest that a change in psychiatrists' current awareness and perceptions is required regarding the importance of impairments in social functioning in patients with schizophrenia.

Psychiatrists concurred that the measurement and recording of social functioning on a regular basis is important, and most assess personal and social functioning at every visit. However, relatively few psychiatrists use specific scales for assessing functioning, with clinical interview being the method of preference, and many respondents also value the opinion of the patient's family. In this survey, the GAF scale [38] was reported as being used most often followed by the PSP scale [39], although other scales (e.g. SOFAS [36], FROGS [37]) are also used in clinical practice. Non-standardized methods of assessment make cross-study comparison difficult, particularly as scales differ in terms of the types of domains assessed, the methods by which information is gathered and the level of complexity of the assessment. An additional complicating factor is the non-concordance between patients' and psychiatrists' assessments of functioning levels [44]. These are amongst several challenges facing psychiatrists when assessing functioning in their daily clinical practice. Others include difficulty in distinguishing functioning deficits from the effects of negative symptoms [7,45] and a frequent lack of distinction between quality of life and functioning measures [7,46]. In addition, only a relatively small number of randomized, controlled clinical trials of antipsychotic agents have assessed social functioning using a specific scale [7]. These aspects have been explored further in a recent research initiative [44,47]. In the current survey, improvements in functioning were normally found to be judged via personal and social relationships or by engagement in activities such as work and study. Although these measures can be considered to be objective and therefore valid and more straightforward to measure, they can also be influenced by external societal factors and individual values. Even when using these ‘objective’ assessment measures, the subjective opinions of the patient, family and psychiatrist will also inevitably form part of the evaluation process [6]. Most participants in this survey agreed that clinical guidelines on social functioning would prove useful to their practice, while opinions were more divided regarding the clinical need to differentiate the assessment of cognitive impairments and social functioning. This supports the suggestions that the current lack of a consensus in the assessment of functioning hinders the effective assessment of patient progress and subsequent improvement of outcomes [18,48].

The results presented here suggest that the current method most commonly used as a primary measure to tackle functioning deficits in patients with schizophrenia is psychosocial intervention. Approximately one third of psychiatrists would reconsider the patient's pharmacological treatment as a first step. The pharmacological strategies most often used were changing the dose of the current antipsychotic or changing the antipsychotic. Respondents appeared to attach only low priority to switching antipsychotic formulation, including oral, oral extended release or long-acting injectable formulations.

## Conclusions

In conclusion, psychiatrists recognize that a large proportion of their patients with schizophrenia have impaired personal and social functioning. Clinicians assess and record functioning on a regular basis, although there is no consensus on how these assessments should be conducted. There was agreement that guidelines on this topic would aid clinical practice. While the majority of psychiatrists would implement psychosocial strategies as an initial step in addressing functioning deficits, a considerable number would choose to alter the patient's pharmacological therapy (although few psychiatrists considered changing the antipsychotic formulation). Most psychiatrists identify improvements in social functioning as a key outcome for patients and their families. The majority also agreed that a distinction between symptom improvement and improvements in social functioning would be clinically useful. Despite this and the literature evidence suggesting a lack of correlation between improving symptoms and improving functioning, enhancing functioning was rated as a less important treatment goal than reducing psychotic symptoms. This may signify a need for increased awareness amongst psychiatrists of the uncertain correlation between improvements in symptoms and in social functioning. These results also highlight that addressing deficits in social and personal functioning in patients with schizophrenia is an important area for further development, where increased emphasis on functioning as a treatment goal may enhance outcomes for patients. It is hoped that the findings of this survey can contribute to the design of activities and tools applicable to everyday clinical practice to improve the assessment and management of social functioning in patients with schizophrenia.

### Limitations

Methodological differences existed between countries in survey distribution. Participants in the survey were selected from those known to the sponsoring pharmaceutical company. However, all surveys were completed anonymously and results collated by an independent organization.

The response rate to the survey was 13% (based on the initial number of surveys produced). As such, the respondents to the survey may have a greater awareness or interest in the topic of functioning and therefore may not fully represent the broader HCP population. Similarly, the extent to which the demographics (in terms of the sex, age and practice setting) of the responders to the survey may systematically differ from psychiatrists across Europe, the Middle East and Africa as a whole, and the potential impact of this on the survey results is not known.

## Competing interests

LH is a full-time employee of Janssen-Cilag Medical and Scientific Affairs Europe, Middle East and Africa. AS is a full-time employee of Janssen-Cilag Medical and Scientific Affairs Europe, Middle East and Africa and a shareholder of Johnson & Johnson. PG, TB, GJ, AR and LS have been members of advisory boards, involved in designing and participating in clinical trials or received educational grants for research, honoraria and travel from Janssen as well as other pharmaceutical companies.

## Authors’ contributions

PG, TB, GJ, AR, LS, LH and AS approved the design and reviewed the results of the survey. All authors developed the draft of the manuscript, participated in subsequent revisions and read and approved the final manuscript.

## Supplementary Material

Additional file 1 **Country of origin of survey respondents. **Summary of the country origin of the survey respondents. Click here for file

Additional file 2 **Respondent demographics. **Overview of the respondent demographics of the survey respondents. Click here for file

Additional file 3 **Summary of survey questions and responses. **Survey questions and responses (% of respondents). Click here for file
