# Supplementary material for: Psychiatrists’ perceptions of the clinical importance, assessment and management of patient functioning in schizophrenia in Europe, the Middle East and Africa
Source: Ann Gen Psychiatry. 2013 Mar 26;12:8. doi: 10.1186/1744-859X-12-8 (PMC3778848; doi:10.1186/1744-859X-12-8)
Supplement: Additional file 1 — Country of origin of survey respondents. Summary of the country origin of the survey respondents. [file 1744-859X-12-8-S1.doc]

Table 1. Country of origin of survey respondents

| **Country** | **Number of respondents** | **Country** | **Number of respondents** |
| --- | --- | --- | --- |
| Austria | 123 | Lebanon | 33 |
| Belgium | 108 | Maghreb countries (Algeria, Morocco, Tunisia) | 673 |
| Croatia | 60 | Malta | 4 |
| Czech Republic | 76 | Nordics (including Norway, Sweden, Finland, Denmark) | 77 |
| Cyprus | 30 | Poland | 71 |
| Egypt | 107 | Portugal | 55 |
| France | 426 | Russia | 199 |
| GCC (Bahrain, Kuwait, Oman, Qatar, Saudi Arabia, UAE) | 148 | Serbia | 74 |
| Germany | 170 | Slovakia | 111 |
| Greece | 165 | Slovenia | 61 |
| Hungary | 262 | South Africa | 59 |
| Iran | 20 | Spain | 110 |
| Iraq | 32 | Switzerland | 132 |
| Israel | 187 | The Netherlands | 93 |
| Italy | 122 | Turkey | 220 |
| Jordan | 50 | UK | 105 |

GCC, Gulf Cooperation Council; UAE, United Arab Emirates
