# Supplementary material for: Psychiatrists’ perceptions of the clinical importance, assessment and management of patient functioning in schizophrenia in Europe, the Middle East and Africa
Source: Ann Gen Psychiatry. 2013 Mar 26;12:8. doi: 10.1186/1744-859X-12-8 (PMC3778848; doi:10.1186/1744-859X-12-8)
Supplement: Additional file 2 — Respondent demographics. Overview of the respondent demographics of the survey respondents. [file 1744-859X-12-8-S2.doc]

Table 2. Respondent demographics

| **Demographic** | | **Respondents (%)** |
| --- | --- | --- |
| Gender | Male | 52 |
|  | Female | 39 |
|  | No response | 9 |
| Length of experience in psychiatry | Less than 10 years | 30 |
|  | 10–19 years | 31 |
|  | 20–29 years | 19 |
|  | More than 29 years | 12 |
|  | No response | 8 |
| Predominant work practice | No response | 58 |
|  | Of those who responded: |  |
|  | Academic | 53 |
|  | Non-academic/other | 47 |
|  | No response | 45 |
|  | Of those who responded: |  |
|  | Privately funded facility | 26 |
|  | Publicly funded facility | 74 |
|  | No response | 25 |
|  | Of those who responded: |  |
|  | Inpatient | 18 |
|  | Outpatient | 31 |
|  | Inpatient and outpatient | 51 |
