# Supplementary material for: Psychiatrists’ perceptions of the clinical importance, assessment and management of patient functioning in schizophrenia in Europe, the Middle East and Africa
Source: Ann Gen Psychiatry. 2013 Mar 26;12:8. doi: 10.1186/1744-859X-12-8 (PMC3778848; doi:10.1186/1744-859X-12-8)
Supplement: Additional file 3 — Summary of survey questions and responses. Survey questions and responses (% of respondents). [file 1744-859X-12-8-S3.doc]

Table 3. Summary of survey questions and responses

| **Survey questions and responses (% of respondents)** | | | | | | | | | | | | |
| --- | --- | --- | --- | --- | --- | --- | --- | --- | --- | --- | --- | --- |
| *Q7. How often do you assess and record personal and social functioning in your patients with schizophrenia? Please select one answer* | | | | | | | | | | | | |
|  | Regularly, at every visit | | | | | | | | | | | 56 |
|  | Every 2–3 visits | | | | | | | | | | | 25 |
|  | 2–3 times a year | | | | | | | | | | | 12 |
|  | Never | | | | | | | | | | | 2 |
|  | No response | | | | | | | | | | | 5 |
| *Q8. Which approach do you use most often to assess your patients’ level of personal and social functioning? Please select one answer* | | | | | | | | | | | | |
|  | Using specifically designed functioning scales | | | | | | | | | | | 6 |
|  | With non-specific scales | | | | | | | | | | | 4 |
|  | I determine it through the clinical interview | | | | | | | | | | | 81 |
|  | I don’t measure my patients’ functionality | | | | | | | | | | | 2 |
|  | No response | | | | | | | | | | | 7 |
| *Q9. If you use a scale to assess your patients’ functioning, which scale do you use most often? Please select one answer* | | | | | | | | | | | | |
|  | Global Assessment of Functioning (GAF) | | | | | | | | | | | 47 |
|  | Personal and Social Performance (PSP) Scale | | | | | | | | | | | 25 |
|  | Social Occupational Functional Scale (SOFAS) | | | | | | | | | | | 9 |
|  | No response | | | | | | | | | | | 19 |
| *Q10. When you detect a low level of social functioning in your patients, what is your first course of action? Please select one answer* | | | | | | | | | | | | |
|  | Use a scale to measure their level of functioning | | | | | | | | | | | 4 |
|  | Use a psychosocial intervention (eg social, psychological, psychoeducation…) | | | | | | | | | | | 41 |
|  | Reconsider their current drug therapy | | | | | | | | | | | 34 |
|  | Seek more family support | | | | | | | | | | | 12 |
|  | No response | | | | | | | | | | | 10 |
| *Q13. Who do you most often ask for support in order to improve social functioning in your patients? Please select one answer* | | | | | | | | | | | | |
|  | Nurse | | | | | | | | | | | 15 |
|  | Family/Caregiver | | | | | | | | | | | 47 |
|  | Psychologist | | | | | | | | | | | 10 |
|  | Social worker | | | | | | | | | | | 14 |
|  | General practitioner | | | | | | | | | | | 1 |
|  | No one | | | | | | | | | | | 3 |
|  | No response | | | | | | | | | | | 11 |
| *Q14. Rate the importance of each of the following factors when selecting a drug therapy to improve your patients’ social functioning* | | | | | | | | | | | | |
|  | | | *Very important* | | *Important* | | *Neutral* | | *Less important* | | *Not important* | *No response* |
| *Efficacy (symptom control)* | | | 75 | | 18 | | 1 | | 0 | | 0 | 6 |
| *Good tolerability* | | | 56 | | 37 | | 1 | | 0 | | 0 | 5 |
| *Non-sedative properties* | | | 20 | | 50 | | 20 | | 4 | | 0 | 6 |
| *Simple dosage* | | | 26 | | 48 | | 15 | | 5 | | 1 | 5 |
| *Improved level of adherence* | | | 41 | | 43 | | 8 | | 2 | | 0 | 5 |
| *Route of administration* | | | 14 | | 43 | | 27 | | 9 | | 2 | 5 |
| *Pharmacokinetic profile* | | | 13 | | 43 | | 27 | | 11 | | 2 | 5 |
| *Potential to preserve/improve cognition* | | | 46 | | 42 | | 6 | | 1 | | 0 | 4 |
| *Q15. During which stage of schizophrenia do you focus most on improving your patients’ social functioning? Please select one answer* | | | | | | | | | | | | |
|  | At onset/less than 6 months since diagnosis | | | | | | | | | | | 41 |
|  | In recently diagnosed patients (less than 5 years) | | | | | | | | | | | 43 |
|  | In chronic patients (with diagnosis longer than 5 years) | | | | | | | | | | | 7 |
|  | At later stages of disorder (established disability) | | | | | | | | | | | 2 |
|  | No response | | | | | | | | | | | 8 |
| *Q17. Please indicate your level of agreement with each of the statements below relating to social functioning in schizophrenia* | | | | | | | | | | | | |
|  | | *Strongly agree* | | *Agree* | | *Neither agree nor disagree* | | *Disagree* | | *Strongly disagree* | | *No response* |
| *A patient’s level of social functioning should be measured regularly and recorded* | | 28 | | 56 | | 11 | | 2 | | 0 | | 3 |
| *A patient’s antipsychotic treatment should be re-evaluated when their everyday social functioning has potential for improvement* | | 32 | | 50 | | 10 | | 4 | | 0 | | 3 |
| *Improvements in tolerability and safety of antipsychotic drugs lead to better patient social functioning* | | 38 | | 45 | | 11 | | 3 | | 0 | | 3 |
| *A patient’s level of social functioning is the best mid- to long-term indicator of their treatment success* | | 33 | | 48 | | 13 | | 3 | | 0 | | 3 |
| *A family member’s opinion on a patient’s level of social functioning is important* | | 29 | | 58 | | 9 | | 1 | | 0 | | 3 |
| *Cognitive impairment and level of social functioning, in patients with schizophrenia, are two separate entities which require two different assessments* | | 18 | | 38 | | 20 | | 17 | | 3 | | 5 |
